# An exosome-derived lncRNA signature identified by machine learning associated with prognosis and biomarkers for immunotherapy in ovarian cancer

**DOI:** 10.3389/fimmu.2024.1228235

**Published:** 2024-02-09

**Authors:** Yongjia Cui, Weixuan Zhang, Wenping Lu, Yaogong Feng, Xiaoqing Wu, Zhili Zhuo, Dongni Zhang, Yichi Zhang

**Affiliations:** ^1^ Guang Anmen Hospital, China Academy of Chinese Medical Sciences, Beijing, China; ^2^ School of Computer and Information Technology, Beijing Jiaotong University, Beijing, China

**Keywords:** exosome-related lncRNA, ovarian cancer, machine learning, prognosis model, immunotherapy response

## Abstract

**Background:**

Ovarian cancer (OC) has the highest mortality rate among gynecological malignancies. Current treatment options are limited and ineffective, prompting the discovery of reliable biomarkers. Exosome lncRNAs, carrying genetic information, are promising new markers. Previous studies only focused on exosome-related genes and employed the Lasso algorithm to construct prediction models, which are not robust.

**Methods:**

420 OC patients from the TCGA datasets were divided into training and validation datasets. The GSE102037 dataset was used for external validation. LncRNAs associated with exosome-related genes were selected using Pearson analysis. Univariate COX regression analysis was used to filter prognosis-related lncRNAs. The overlapping lncRNAs were identified as candidate lncRNAs for machine learning. Based on 10 machine learning algorithms and 117 algorithm combinations, the optimal predictor combinations were selected according to the C index. The exosome-related LncRNA Signature (ERLS) model was constructed using multivariate COX regression. Based on the median risk score of the training datasets, the patients were divided into high- and low-risk groups. Kaplan-Meier survival analysis, the time-dependent ROC, immune cell infiltration, immunotherapy response, and immune checkpoints were analyzed.

**Results:**

64 lncRNAs were subjected to a machine-learning process. Based on the stepCox (forward) combined Ridge algorithm, 20 lncRNA were selected to construct the ERLS model. Kaplan-Meier survival analysis showed that the high-risk group had a lower survival rate. The area under the curve (AUC) in predicting OS at 1, 3, and 5 years were 0.758, 0.816, and 0.827 in the entire TCGA cohort. xCell and ssGSEA analysis showed that the low-risk group had higher immune cell infiltration, which may contribute to the activation of cytolytic activity, inflammation promotion, and T-cell co-stimulation pathways. The low-risk group had higher expression levels of PDL1, CTLA4, and higher TMB. The ERLS model can predict response to anti-PD1 and anti-CTLA4 therapy. Patients with low expression of PDL1 or high expression of CTLA4 and low ERLS exhibited significantly better survival prospects, whereas patients with high ERLS and low levels of PDL1 or CTLA4 exhibited the poorest outcomes.

**Conclusion:**

Our study constructed an ERLS model that can predict prognostic risk and immunotherapy response, optimizing clinical management for OC patients.

## Introduction

Global Cancer Statistics reports that ovarian cancer (OC) caused the death of 207,252 individuals worldwide in 2020 (1). OC has the highest mortality rate among gynecological malignancies (2). Aggressive first-line treatment with surgery and adjuvant chemotherapy is the main treatment for advanced OC, but within 2-3 years after diagnosis, 70% of patients with advanced-stage OC still have a relapse (3, 4). The introduction of anti-VEGF and PARP inhibitors as treatment modalities has significantly increased the duration of progression-free survival (PFS) for recurrent OC patients, although progression remains unavoidable in most cases of OC patients. In the last decade, accumulating studies have revealed that immune checkpoint inhibitors (ICIs) have revolutionized the treatment of multiple cancers. However, the effect of immunotherapy on the clinical treatment of OC is not satisfactory (5–7), only 8 to 9.6% of OC patients benefit from ICI therapy (8), especially in patients with PD-1, PD-L1, or CTLA4 negative patients. The limited benefit of immunotherapy has led researchers to develop new biomarkers to predict the efficacy of OC immunotherapy to improve prognosis.

The tumor immune microenvironment (TIME) is considered a critical factor in the efficacy of immune therapy against cancer (9). The TIME refers to the immune infiltrating microenvironment, which consists of a large number of immune cells gathered in and around the tumor (10). Immune cells in the TIME, including T cells, B cells, natural killer cells, macrophages, etc., participate in immune surveillance and anti-tumor responses through various mechanisms such as releasing cytotoxic molecules, producing cytokines, and regulating immune responses. Nevertheless, tumor cells can escape immune cell attack by activating immune checkpoints. Immune checkpoints include PD-L1, CTLA-4, and others. Tumor cells release exosomes that serve as mediators for immune escape and influence the efficacy of immune therapy (11–13).

Exosomes, small membrane vesicles ranging in size from 30 to 150 nm, are produced by various cells. They play a crucial role in mediating intercellular communication and transporting cargo molecules, including proteins, DNA, RNA, microRNA, and lncRNA. This has garnered significant interest among researchers (14). A substantial quantity of exosomes can be found in the blood and ascites of OC patients. These exosomes have been associated with OC progression and its treatment, spanning various aspects (15–20) including immunotherapy (21, 22), angiogenesis (14, 23), chemotherapy resistance (24, 25), and tumor metastasis (26, 27). They hold promise as potential diagnostic and prognostic biomarkers. Long non-coding RNA (lncRNA) is characterized as non-coding RNA with a length exceeding 200 nucleotides, constituting approximately 3% of the total RNA content within exosomes (28). Furthermore, increasing evidence suggests that epigenetic regulation of lncRNA plays a significant role in reprogramming the phenotype of immune cells in TIME, particularly in OC. For example, SNHG12 enhances immune escape by promoting the IL-6/miR-21 crosstalk between OC cells and M2 macrophages, leading to increased expression of PD-L1 (29). LncRNA PVT1 combined with PD-1 inhibitors can inhibit the progression of OC in treatment (30). Accumulating evidence suggests that epigenetic regulation of exosome-derived lncRNA plays an important role in OC by reprogramming the phenotype of immune cells in TIME (31). However, previous studies only evaluated the prognosis of OC based on exosome-related genes prediction models (32) and did not integrate the necessary information about exosome lncRNA. Previous studies have demonstrated the effectiveness of exosome-related lncRNA prognostic models in breast cancer (33), esophageal squamous cell carcinoma (34), and hepatocellular carcinoma (35, 36). However, their applicability in OC remains uncertain. At present, machine learning is widely used in constructing predictive models for tumor prognosis, treatment, and diagnosis (37–40). However, the prediction model of exosome-related genes is based only on the Lasso algorithm (32), which is not robust.

In our paper, taking into account the complex role of exosomes, we aimed to integrate and develop the exosome-related lncRNA signature (ERLS) to improve outcomes for OC patients. Specifically, we construct a more robust ERLS model by using 10 machine learning algorithms and their 117 combinations, which were trained based on the 10-fold cross-validation framework. Subsequently, OC patients were divided into high- and low-risk groups based on their ERLS risk scores, and the characteristics of immune cell infiltration, immunotherapy response, and immune checkpoint were also identified. This work may help optimize immune therapy and further improve clinical outcomes in patients with OC.

## Materials and methods

### Data downing and processing

RNA sequencing expression data for tumor tissues from 420 patients with OC and their corresponding clinical information, were obtained from The Cancer Genome Atlas (TCGA). (https://portal.gdc.cancer.gov/projects/TCGA-OV). The RNA seq transcripts per kilobase million (TPM) including the expression of 16901 lncRNA and 19962 protein-coding genes were downed and further log-2 transformed. However, complete clinical data (including age, stage, grade, and tumor_residual) were available for 369 patients (**Table 1**). Due to missing values in the clinical information, 51 patients were excluded from the time-dependent ROC analysis. For other analyses, we used the RNA sequencing expression data of 420 patients. The GSE102073 dataset was downloaded from GEO (https://www.ncbi.nlm.nih.gov/geo/) as an external validation of the accuracy of the ERLS model. In addition, 121 exosome-related genes were obtained from the ExoBCD database (https://exobcd.liumwei.org/), which were summarized in **Supplementary Data 1**. The expression data of lncRNA in normal ovarian tissue were obtained from the GTEx database (https://www.gtexportal.org/home/downloads/adult-gtex#bulk_tissue_expression).

**Table 1:** Summary of clinical information for patients with OC.

	Train datasets (n=263)	validation datasets (n=106)
Age(year)	59.80 ± 11.55	59.65 ± 11.31
Stage		
I	0	0
II	14	5
III	208	85
IV	41	16
Grade		
1	0	0
2	26	16
3	236	90
4	1	0
Tumor_residual		
No macroscopic disease	49	26
1-10 mm	142	52
11-20 mm	19	7
>20 mm	53	21

### Screening of candidate exosome-related lncRNAs

The rcorr function in the Hmisc package of R calculated the Pearson correlation coefficients to determine the correlation between exosome-related genes expression and the corresponding lncRNAs. Subsequently, exosome-related lncRNAs were selected according to the criteria of p< 0.05 and |Cor|> 0.4. Meanwhile, the survfit function in the survival package of R was used to perform a univariate Cox regression to identify prognostic lncRNAs with a significant p threshold of 0.05. Finally, lncRNAs that overlap with exosome-related lncRNAs and prognostic lncRNAs were selected as candidate lncRNAs for the machine learning process.

### Identification of exosome-related lncRNA signature (ERLS) based on machine learning

The 420 OC patients from the TCGA cohort were divided in a 7:3 ratio into training and validation datasets using the createDataPartition function in the caret package. To identify potential biomarkers for OC, candidate lncRNAs were further screened using 10 machine learning algorithms and 117 algorithm combinations. In the training datasets, 10 machine learning algorithms and 117 algorithm combinations were employed to identify the optimal algorithm combinations based on a 10-fold cross-validation, which was verified in the verification data set. The selection of the best algorithm combinations was based on Harrell’s consistency index (C index) in the validation datasets. 10 machine learning algorithms include Random Survival Forest (RSF), Lasso, Elastic Net (Enet), Ridge, Generalized Boosted Regression (GBM), Stepwise Cox, CoxBoost, Cox Partial Least Squares Regression (plsRcox), Supervised Principal Components (SuperPC), and survival support vector machine (survival-SVM). Subsequently, the selection of important variables based on the optimal algorithm combinations was achieved using the stepAIC function in the MASS package. The Akaike Information Criterion (AIC) is used to compare models, which takes into account the statistical fit of the models and the number of variables used for the fit. The regression model with a small AIC value should be selected first, which shows that the model has obtained a sufficient fitting degree with few parameters. See the **Methods Supplement** for more details. Finally, we constructed the ERLS model using a multivariate COX regression, and the risk score was constructed with the following formula: $\text{Risk score}=\sum_{\text{i}=1}^{\text{n}}(\text{coefi }*\text{ Expi})$ , Expi indicated the expression level for each exosome-related lncRNA, and Coei indicated the corresponding Cox regression coefficient.

Afterward, we proceeded to validate the prognostic value of the ERLS model across multiple datasets, including the validation datasets, the entire TCGA cohort, and the GSE102073 dataset. Initially, patients were divided into high- and low-risk groups based on the median risk score of the training datasets. This same risk stratification was applied to the validation datasets, the entire TCGA cohort, and the GSE102073 dataset. Subsequently, survival analysis and time-dependent ROC curves were conducted to evaluate the predictive accuracy of the ERLS model in these datasets. Additionally, univariate and multivariate COX regression analyses were performed to assess the prognostic impact of the ERLS compared to other clinical factors in OC patients.

### Evaluating the immune cell infiltration in OC patients

Infiltration levels of 28 types of immune cells were calculated using the R package ssGSEA (33, 34). The set of genes for the 28 immune cell markers was downloaded from the TISIDB database (http://cis.hku.hk/TISIDB/) (41). Additionally, we also used the R package xCell to analyze and evaluate the infiltration ratios of 64 cell types in the high-risk and low-risk groups. The xCell R package, which is based on the ssGSEA method, can perform an immune infiltration analysis based on the gene expression data for 64 immune and stromal cells (42). Finally, we used the limma package to analyze differential gene expression between the high-risk and low-risk groups. Subsequently, the GSEA package performed an enrichment analysis of differential immune genes to investigate the difference in the immune function in the high-risk and low-risk groups. The R package “clusterProfiler” was used to conduct Gene Ontology (GO), Kyoto Encyclopedia of Genes and Genomes (KEGG) on the different genes in the high-risk and low-risk groups.

### Predicting immunotherapy response based on the ERLS model

The ERLS model can identify different survival risks and immune microenvironmental characteristics in OC patients. Next, we focused on the ability of the ERLS model to discriminate response to immunotherapy. We evaluated immunotherapy response based on The Tumor Immune Dysfunction and Exclusion (TIDE) (http://tide.dfci.harvard.edu/), The Cancer Immunome Atlas (TCIA) (https://tcia.at/home), Tumor Mutation Burden (TMB), and the expression of immune checkpoint in high-risk and low-risk groups. The TIDE Tool was used to assess the potential for tumor immune escape of tumor samples with gene expression profiles and predict response rate to immune checkpoint blockade (ICB) (43). The effectiveness of immunotherapy was lower with higher TIDE scores. The immunophenoscore (IPS) was obtained from the TCIA database to evaluate the benefit of anti-PD1 and anti-CTLA4 immunotherapy (44). The higher the IPS score, the more sensitive the response to immunotherapy. TMB is an indicator for evaluating the frequency of gene mutations. The more tumor gene mutations, the higher the number of antigens on the cell surface, and the greater the benefit of immunotherapy. The expression of immune checkpoints including PD1, PDL1, and CTLA4, was significantly correlated with the efficacy of immunotherapy. Therefore, we focused on differences in the expression of immune checkpoints in the two groups.

### Exosome isolation and real-time quantitative PCR

We used the limma package to compare the expression of lncRNA in normal ovarian tissue and OC tissue. We found that the expression of lncRNAs showed differential patterns (**Supplementary Figure 1**). Next, we detected the expression of some lncRNAs in exosomes from SKOV3 cells, IOSE80 cells, and OVCAR8 cells. SKOV3 cells, IOSE80 cells, and OVCAR8 cells were obtained from Procell Life Science & Technology Co. Ltd. (Wuhan, China). Firstly, SKOV3 cells were cultured in McCoy’s 5A medium with 10% fetal bovine serum (FBS, Gibco, 10099141) and 1% penicillin-streptomycin (Gibco, 10378016) at 37 °c and 5% CO2. IOSE80 cells and OVCAR8 cells were cultured in RPMI-1640 medium with 10% FBS and 1% penicillin-streptomycin at 37 °c and 5% CO2. Secondly, when cell fusion reached 70%-80%, washing with phosphate-buffered saline (PBS) 3 times, they were cultured in the basic medium. After 48 h culture, the conditioned media were collected. Exosomes were extracted from the conditioned media of SKOV3 cells, IOSE80 cells, and OVCAR8 cells using ultracentrifugation. We used three methods to identify exosomes, including the transmission electron microscope (TEM), the nanoparticle tracking analysis (NTA), and Western blot (WB). Subsequently, we performed real-time quantitative PCR detection of lncRNA in exosomes. We isolated RNA using TRIZOL (Invitrogen, 10296028). RNA was reversed to cDNA using SuperScript™ III First-Strand Synthesis SuperMix for qRT-PCR (Invitrogen, 11752050). Then, according to the manufacturer’s instructions, we performed RT-qPCR using SYBR™ Select mix (ABI-invitrogen, 4472920). The AC134312.1 primers used were TCTTCACCCATGTCCTGTGC (forward primer) and CAGGGGTCCTTCTGTTCGTC (reverse primer). The PCOLCE.AS1 primers used were TTGGCCACTGTGACCTGTTC (forward primer) and CTGAGCTAGAACCCAGGAGC (reverse primer). The LEMD1.AS1 primers used were CCACTGGTAACTTGCCGTCT (forward primer) and AAATGCCCTTCTCCTGTCGG (reverse primer). The LINC00892 primers used were GGATGTTCTTTGCTGGGCTG (forward primer) and ATCAAGCTGCCTCTCGGAAG (reverse primer). The AC010834.3 primers used were GCCTGTTCACACATTGCTGG (forward primer) and CCTTGGGCTCACCCATGATT (reverse primer). The AL138820.1 primers used were GTTATTGGGCTTGCTGCTGG (forward primer) and TTCAGGGAAGAGGTGCCATC (reverse primer). The relative expression levels were calculated using the 2-ΔΔCt method. The RT-PCR and WB experiments were independently repeated three times, with three replicate wells for each independent repetition.

### Data analysis

Data processing and statistical analysis were performed with R software (version 4.2.2) (https://cran.r-project.org/). Pearson correlation coefficients were calculated using the Hmisc package. Kaplan–Meier (KM) survival analysis, univariate and multivariate Cox regression analyses were performed using the survival package. Machine learning was carried out using the glmnet, randomForestSRC, CoxBoost, plsRcox, superpc, gbm, survivalsvm, and MASS packages. The time-dependent ROC curves were generated using the timeROC package. Violin plots were generated using the VioPlot package. Immune cell infiltration analysis was carried out with the ssGSEA and xCell package. Differential gene expression analysis was performed using the limma package. The GSEA package conducted an enrichment analysis of differential gene expression. The “clusterProfiler” package was utilized for Gene Ontology (GO) and Kyoto Encyclopedia of Genes and Genomes (KEGG) analyses. PCR results were drawn using GraphPad Prism. *P< 0.05, **P< 0.01, ***P< 0.001, ****P< 0.0001.

## Results

### Workflow

**Figure 1:**
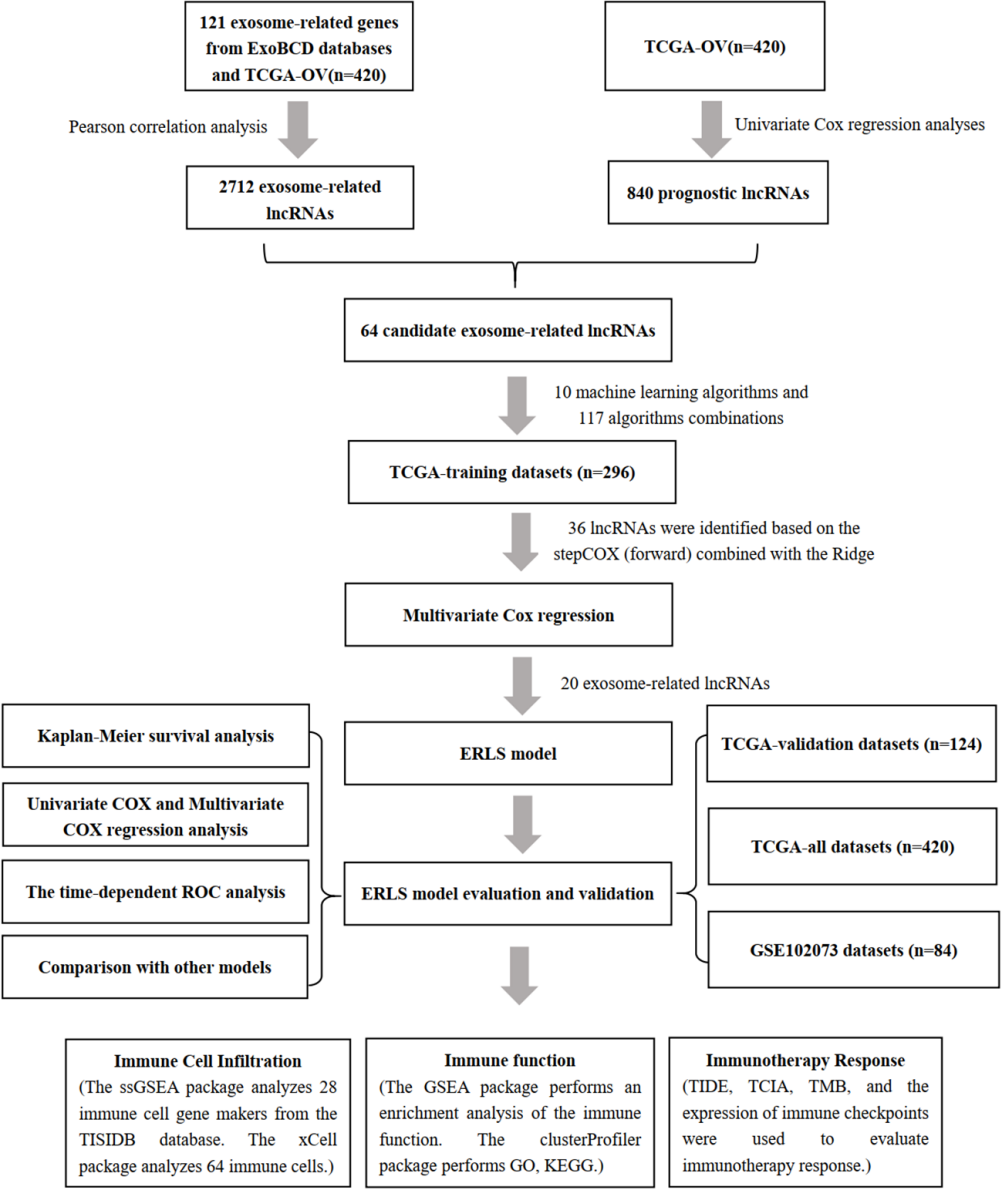
Workflow for constructing the ERLS model.

As shown in **Figure 1**, we constructed the ERLS model according to the following process.

### Screening of candidate exosome-related lncRNAs

**Figure 2:**
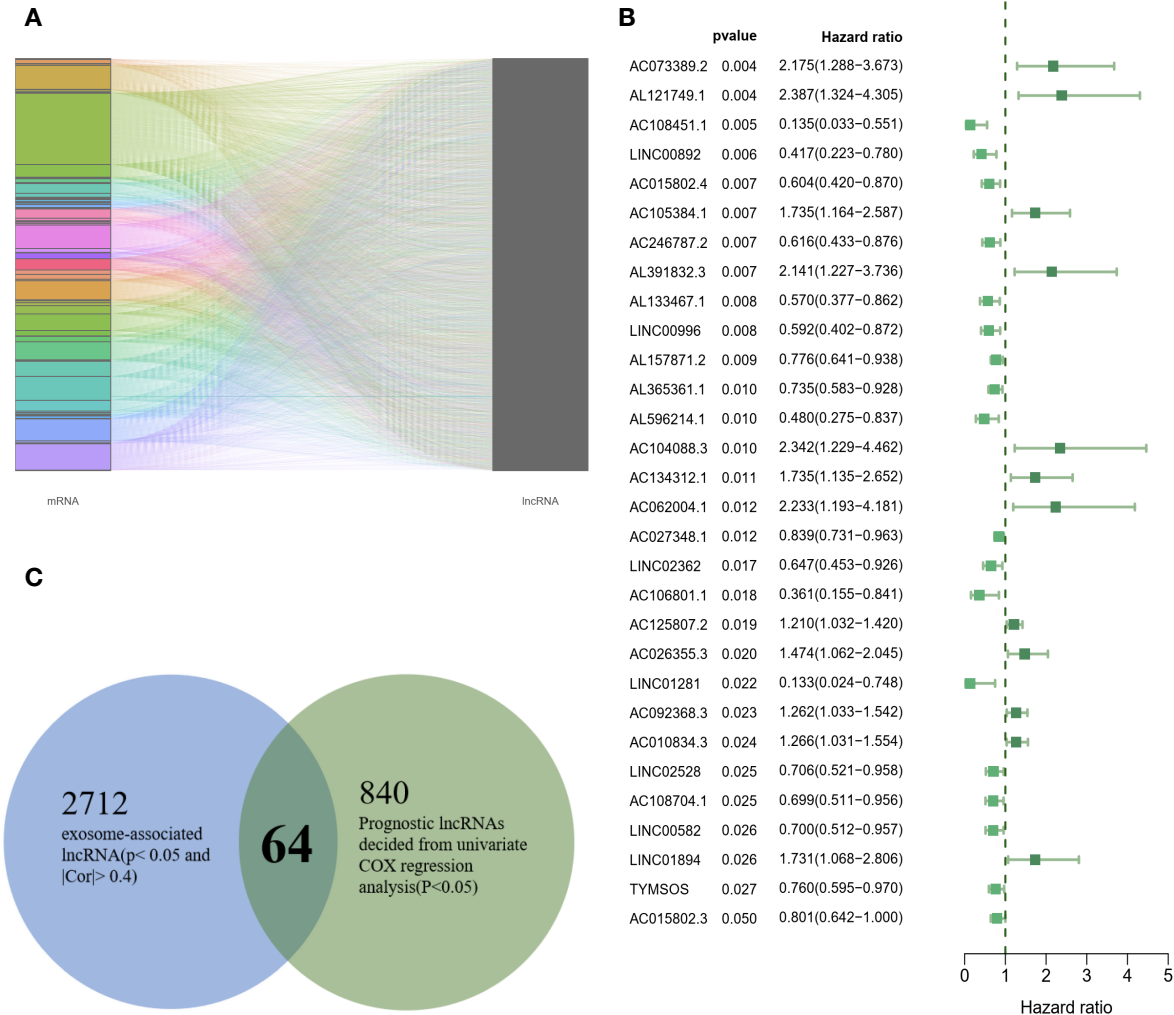
64 candidate exosome-related lncRNAs. **(A)** A total of 2712 exosome-related lncRNAs (|Cor|≥0.4 and P<0.05). **(B)** 30 randomly selected lncRNAs were visualized in 840 lncRNAs. **(C)** 64 lncRNAs were incorporated into subsequent machine learning.

According to the setting |Cor|≥0.4 and P<0.05, a total of 2712 exosome-related lncRNAs were found (**Figure 2A**), and the specific correspondences between lncRNAs and mRNAs were shown in **Supplementary Data 2**. In addition, the 840 lncRNAs were identified as having significant prognostic values with univariate COX regression analysis (**Figure 2B**), the detailed information on lncRNAs was illustrated in **Supplementary Data 3**. Finally, a set of 64 lncRNAs was subjected to a subsequent machine learning process to construct an exosome-related lncRNA signature (ERLS) (**Figure 2C**).

### Establishment of exosome-related lncRNA signature (ERLS) based on machine learning

**Figure 3:**
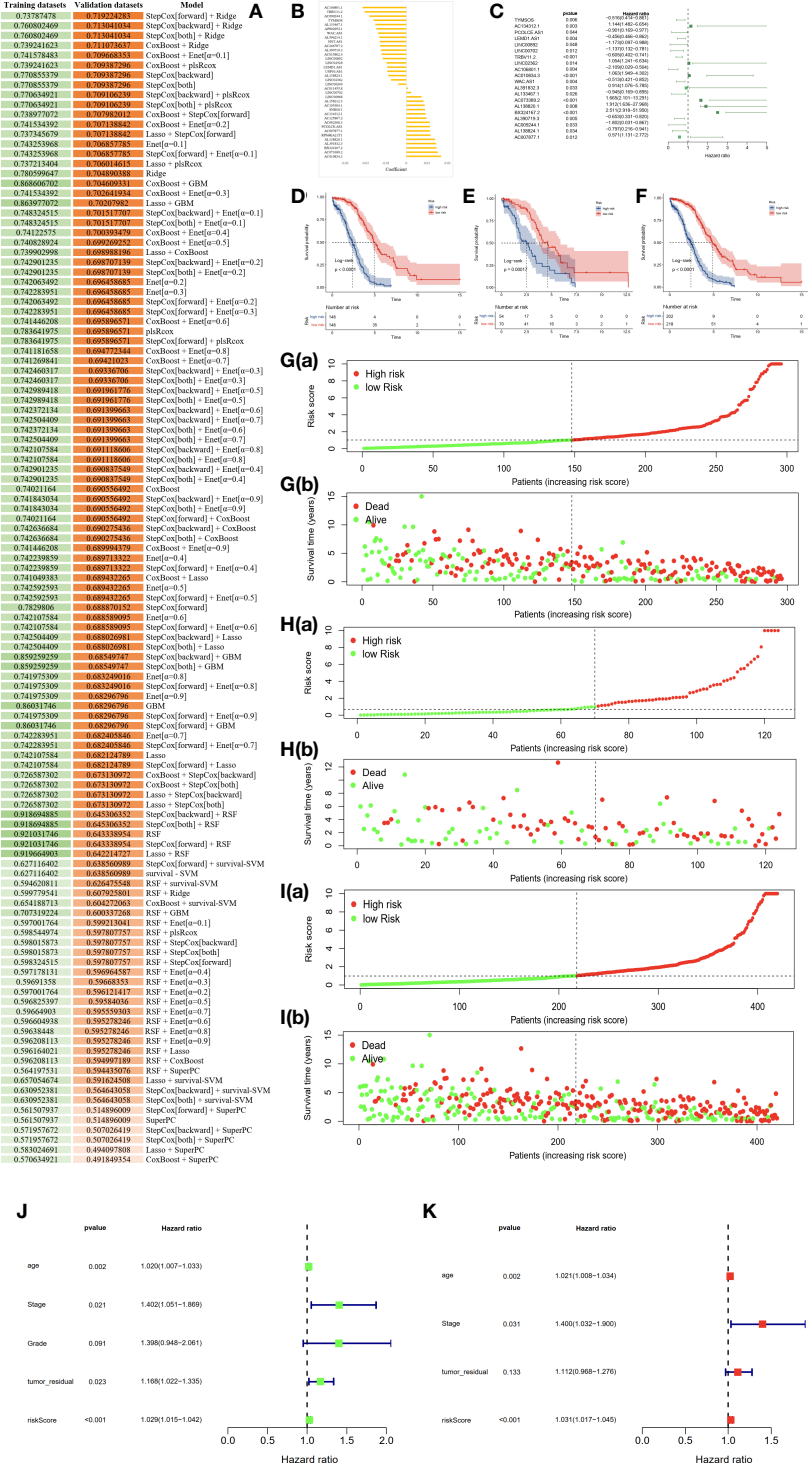
An ERLS was identified based on 10 machine learning algorithms and its clinical prognostic value. **(A)** A total of 117 algorithm combinations based on 10-fold cross-validation, the C-index of each model was calculated in the validation datasets. **(B)** 36 lncRNAs and their coefficients were identified based on the stepCOX (forward) combined with the Ridge algorithm. **(C)** Multivariate Cox regression analysis screened out 20 exosome-related lncRNAs that were independently associated with OS. **(D–F)**. Kaplan-Meier survival analysis in the training datasets, validation datasets, and the entire TCGA cohort. **(G–I)** The risk score curve and the survival state heat map in the training datasets, validation datasets, and the entire TCGA cohort. **(J)** Univariate COX regression analysis of clinical factors and the ERLS for OS. **(K)** Multivariate COX regression analysis of clinical factors and the ERLS for OS.

To establish an exosome-related lncRNA signature (ERLS) based on machine learning, the RNA sequencing expression data of 420 patients with OC were randomly divided into training datasets and validation datasets according to the 7:3 ratio. The training datasets included 296 patients, and the validation datasets included 124 patients. In the training datasets, we integrated 10 machine learning algorithms and 117 algorithm combinations based on the 10-fold cross-validation framework to select important lncRNA and calculate the C-index of each model in the validation datasets. The stepCox (forward) algorithm combined with the Ridge algorithm showed the highest C-index (0.7192), which was determined as the optimal model (**Figure 3A**), see **Supplementary Data 4** for details. With the stepCOX (forward) combined with Ridge algorithm analysis, based on the smallest AIC area, we identified 36 important lncRNAs (**Figure 3B**). We used multivariate Cox regression analysis to select 20 exosome-related lncRNAs that were independently associated with overall survival (OS) (**Figure 3C**; **Table 2**). These 20 lncRNAs were used to develop an ERLS model that evaluated the prognostic risk of OC patients. The ERLS model was constructed using the following formula:(-0.5161*the expression of TYMSOS)+(1.1441*the expression of AC134312.1)+(-0.9014*the expression of PCOLCE.AS1)+(-0.456*the expression of LEMD1.AS1)+(-1.1728*the expression of LINC00892)+(-1.1375*the expression of LINC00702)+(-0.6047*the expression of TRBV11.2)+(1.0543*the expression of LINC02362)+(-2.1089*the expression of AC106801.1)+(1.0632*the expression of AC010834.3)+(-0.5132*the expression of WAC.AS1)+(0.9141*the expression of AL391832.3)+(-0.9446*the expression of AL133467.1)+(1.6647*the expression of AC073389.2)+(1.9116*the expression of AL138820.1)+(2.5106*the expression of BX324167.2)+(-0.6528*the expression of AL390719.3)+(-1.8016*the expression of AC009244.1)+(-0.7968*the expression of AL138824.1)+(0.5713*the expression of AC007877.1).

**Table 2:** 20 exosome-related lncRNA signature in the ERLS.

	coef	HR	95%CI	P-value
TYMSOS	-0.5161	0.5968	(0.41389,0.8607)	0.005721
AC134312.1	1.1441	3.1398	(1.48162,6.6536)	0.002827
PCOLCE.AS1	-0.9014	0.406	(0.16866,0.9773)	0.044301
LEMD1.AS1	-0.456	0.6338	(0.46622,0.8617)	0.003616
LINC00892	-1.1728	0.3095	(0.09693,0.9883)	0.047717
LINC00702	-1.1375	0.3206	(0.13159,0.7812)	0.012302
TRBV11.2	-0.6047	0.5463	(0.40245,0.7414)	0.000105
LINC02362	1.0543	2.8699	(1.24144,6.6344)	0.013671
AC106801.1	-2.1089	0.1214	(0.02922,0.5042)	0.003702
AC010834.3	1.0632	2.8958	(1.94921,4.3019)	1.4E-07
WAC.AS1	-0.5132	0.5986	(0.42082,0.8515)	0.004313
AL391832.3	0.9141	2.4946	(1.07567,5.7855)	0.033179
AL133467.1	-0.9446	0.3888	(0.16891,0.8951)	0.026388
AC073389.2	1.6647	5.2842	(2.10093,13.2907)	0.000404
AL138820.1	1.9116	6.7636	(1.63566,27.9681)	0.008307
BX324167.2	2.5106	12.3125	(2.91812,51.9504)	0.000631
AL390719.3	-0.6528	0.5206	(0.3306,0.8198)	0.00484
AC009244.1	-1.8016	0.165	(0.03143,0.8665)	0.033233
AL138824.1	-0.7968	0.4508	(0.21586,0.9414)	0.033954
AC007877.1	0.5713	1.7705	(1.13104,2.7716)	0.012471

HR hazard ratio, CI confidence interval.

Next, a risk score was calculated using the predict function within R software in the training datasets. Patients were divided into high- and low-risk groups based on the median risk scores in the training datasets. The threshold was then extended to the validation datasets. Subsequently, Kaplan-Meier survival analysis was employed to evaluate the differences in OS between the high-risk and low-risk groups, with the results indicating a significant reduction in OS for patients in the high-risk group (**Figures 3D–F**). The figures of the risk score curve and the survival state heat map for the training and validation datasets were shown in **Figures 3G–I**. Furthermore, we performed a univariate COX regression analysis on the risk score, stage, grade, age, and tumor_residual. Finally, the risk score, age, stage, age, and tumor_residual were selected for multivariate Cox regression analysis, which revealed that the risk score and stage were independent prognostic factors for OC (**Figures 3J, K**).

**Figure 4:**
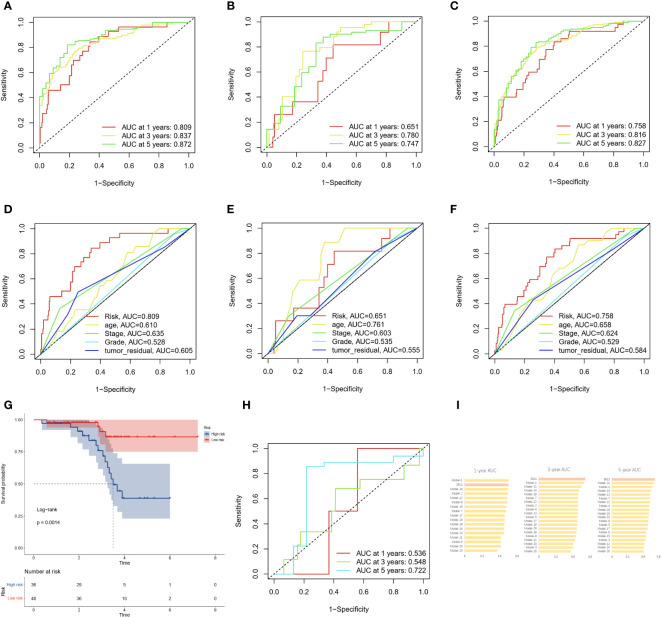
The AUC and validation of the ERLS in OC patients. **(A–C)** The AUC of the ERLS in the training datasets, validation datasets, and the entire TCGA cohort. **(D–F)** The AUC of the ERLS and clinical characteristics in the training datasets, validation datasets, and the entire TCGA cohort. **(G)** Kaplan-Meier survival analysis in the GSE102073 dataset (log-rank test: P=0.0014). **(H)** The AUC of the ERLS in the GSE102073 dataset. **(I)** Comparison of AUC on the ERLS with other models in the entire TCGA cohort.

The area under the curve (AUC) of the time-dependent ROC analysis for 1-year, 3-year, and 5-year was 0.758, 0.816, and 0.827 for patients in the entire TCGA cohort, respectively, indicating that the ERLS model has a certain accuracy in predicting OS in OC patients (**Figures 4A–C**). We incorporated the clinical characteristics of age, stage, age, tumor residual, and the ERLS into the time-dependent ROC analysis and found that the AUC of the ERLS remained always larger than other clinical characteristics, which was 0.809, 0.651, and 0.758, respectively (**Figures 4D–F**). Furthermore, external validation was performed using the GSE102073 datasets, in which only 11 lncRNAs (PCOLCE-AS, TYMSOS, LEMD1-AS1, LINC00892, LINC00702, LINC02362, AC010834.3, WAC-AS1, AL391832.3, AC073389.2, and AC009244.1) were covered in the ERLS. Based on the median risk score derived from the training datasets, the patients were divided into high- and low-risk groups. The ERLS showed a significant discriminatory ability in predicting the prognosis of the two groups (**Figure 4G**). The AUC of the time-dependent ROC values for 1-year, 3-year, and 5-year survival was 0.536, 0.548, and 0.722, respectively (**Figure 4H**).

We also compared the AUC values of the ERLS at 1-year, 3-year, and 5-year in the entire TCGA cohort with 21 other previously published prognostic features for OC patients (see **Supplementary Data 5**). These 21 prognostic features are related to N6-methyladenosine, cell apoptosis, autophagy, immunity, mitochondria, and others. The results showed that the ERLS is competitive among these models (**Figure 4I**).

### Assessing the immune cell infiltration based on the ERLS model

**Figure 5:**
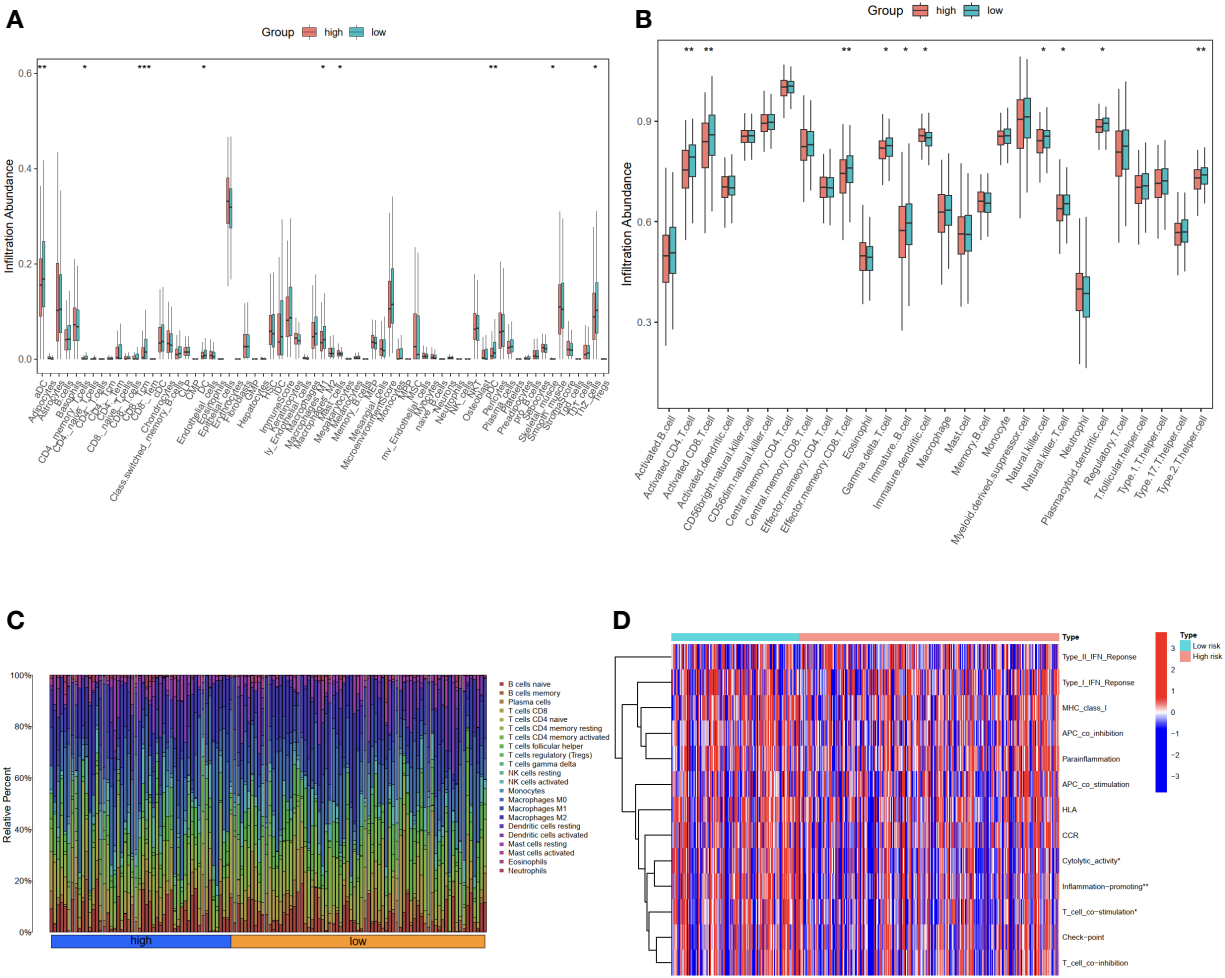
Evaluation of immune cell infiltration in high-risk and low-risk groups using xCell and ssGSEA. **(A)** The proportion of 64 cells in the high-risk group compared to the low-risk group was based on the xCell packages. **(B)** The proportion of immune cells in the high-risk group compared to the low-risk group based on the ssGSEA packages. **(C)** The proportion of immune cells in each OC patient. **(D)** The differential immune functions in the high-risk and low-risk groups. *P<0.05; **P<0.01; ***P<0.001.

To better understand the characteristics of the immune microenvironment between the high-risk group and the low-risk group, the xCell and ssGSEA packages were employed to investigate the proportion of immune cells. Using the xCell R package, we found that the low-risk group demonstrated higher levels of aDC, CD4 memory T cells, CD8 T cells, DC, M1 macrophages, mast cells, pDC, skeletal muscle, and Th2 cells (**Figure 5A**). Subsequently, the ssGSEA analysis further confirmed that the low-risk group was associated with higher infiltration of activated CD4 T cell, activated CD8 T cell, effector memory CD8 T cell, immature B cell, gamma delta T cell, natural killer cell, natural killer T cell, plasmacytoid dendritic cell, Type 2 T helper cell, in addition to immature dendritic cell (**Figure 5B**). The proportion of immune cells in each OC patient is shown in **Figure 5C**. The observed differences in immune cell infiltration between the high-risk and the low-risk groups may be contributed to cytolytic activity, inflammation promoting, and T-cell co-stimulation pathways (**Figure 5D**).

**Figure 6:**
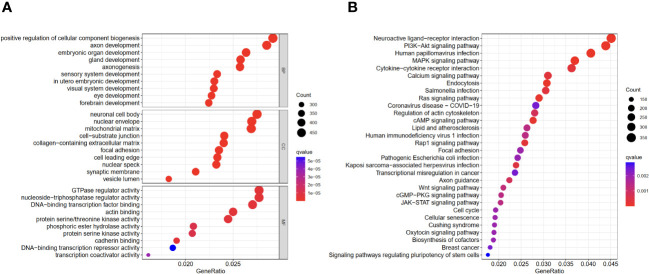
GO and KEGG enrichment analyses of different expressed genes in high- and low-risk groups. **(A)** GO results. **(B)** KEGG results.

In order to understand the different immune functions of the high-risk group and the low-risk group, we performed a Gene ontology (GO) and Kyoto Encyclopedia of Genes and Genomes (KEGG) enrichment analysis. GO results showed that different expressed genes were mainly involved in the biological process (BP) of positive regulation of cellular component biogenesis, embryonic organ development, and axonogenesis. In cellular components (CC), they were related to the cellular mitochondrial matrix and cell-substrate junction, while molecular functions (MF) mainly regulate GTPase regulator activity. The results of the KEGG analysis showed that the different expressed genes were mainly involved in the MAPK signaling pathway and the PI3K-Akt signaling pathway (**Figures 6A, B**).

### The immunotherapy response on different groups

**Figure 7:**
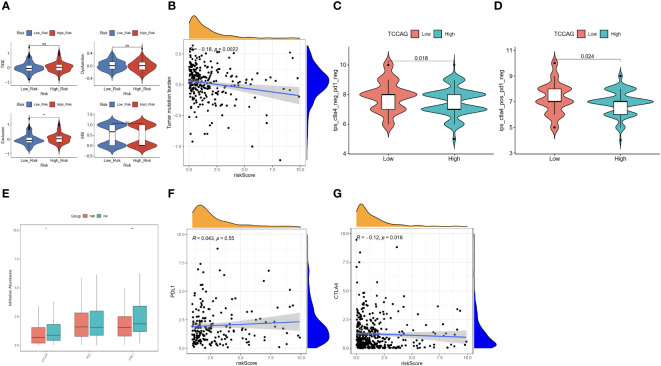
Evaluation of the immunotherapy response based on the ERLS model. **(A)** The TIDE score, the Exclusion score, the MSI, and the Dysfunction score. **(B)** Pearson’s correlation analysis between TMB and risk score. **(C, D)** The immunotherapy response for PD1 or CTLA4 between the high-risk group and the low-risk group. **(E)** The different expressions of PDL1, PD1, and CTLA4 between the high-risk group and the low-risk group. **(F, G)** Pearson’s correlation analysis between the expression of PDL1 or CTLA4 and the risk score. “ns” represents “not significant”. *P < 0.05, **P < 0.01.

Differences in immune cell infiltration can lead to differences in response to immunotherapy. Therefore, we explored the value of the ERLS in immunotherapy. A significant difference in the Exclusion score was found between the high-risk and low-risk groups, but not in the TIDE score, the dysfunction score, and the MSI score. Notably, a trend toward higher TIDE scores and lower MSI scores was observed in the high-risk group compared to the low-risk group (**Figure 7A**). In addition, we observed an inverse association between tumor mutational burden (TMB) and the ERLS risk score, which may suggest that the high-risk group has less benefit from immunotherapy (**Figure 7B**). The potential of the ERLS model to respond to anti-PD1 and anti-CTLA4 immunotherapy was further assessed in the TICA database. As shown in **Figures 7C, D**, the ERLS model could identify the response to anti-PD1, anti-CTLA4, or their combination. Furthermore, we found that the low-risk group had higher expression of PDL1 or CTLA4 (**Figure 7E**). The risk score of the ERLS was negatively correlated with CTLA4 expression, and no significant association was found between the expression of PDL1 and the risk score (**Figures 7F, G**). These findings provided some evidence for the predictive ability of the ERLS model to identify responses to immunotherapy.

**Figure 8:**
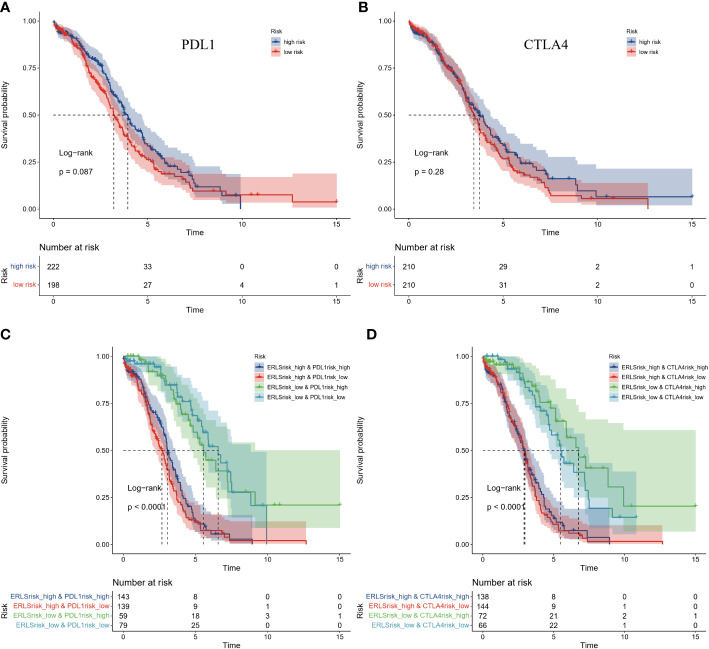
The comparison of Kaplan-Meier survival analysis based on combined ERLS with PDL1 or CTLA4. **(A, B)** The Survival analysis for the expression of PDL1 or CTLA4. **(C, D)** The survival analysis was based on combined ERLS with PDL1 or CTLA4.

However, we have observed that the gene expression of PDL1 and CTLA4 did not seem to distinguish the prognosis risk between the high-risk group and the low-risk group. The two groups were divided according to the median expression of PDL1 and CTLA4 in the training datasets (**Figures 8A, B**). Subsequently, a survival comparison was performed among four groups of OC patients who were identified based on combined ERLS with PDL1 or CTLA4. The results of this comparison revealed that the ERLS was able to differentiate the outcomes of patients with similar PDL1 or CTLA4 levels. Patients with low expression of PDL1 or high expression of CTLA4 and low ERLS exhibited significantly better survival prospects compared to the other three groups, whereas patients with high ERLS and low levels of PDL1 or CTLA4 exhibited the poorest results relative to the other groups (**Figures 8C, D**).

### Exosome isolation and real-time quantitative PCR

**Figure 9:**
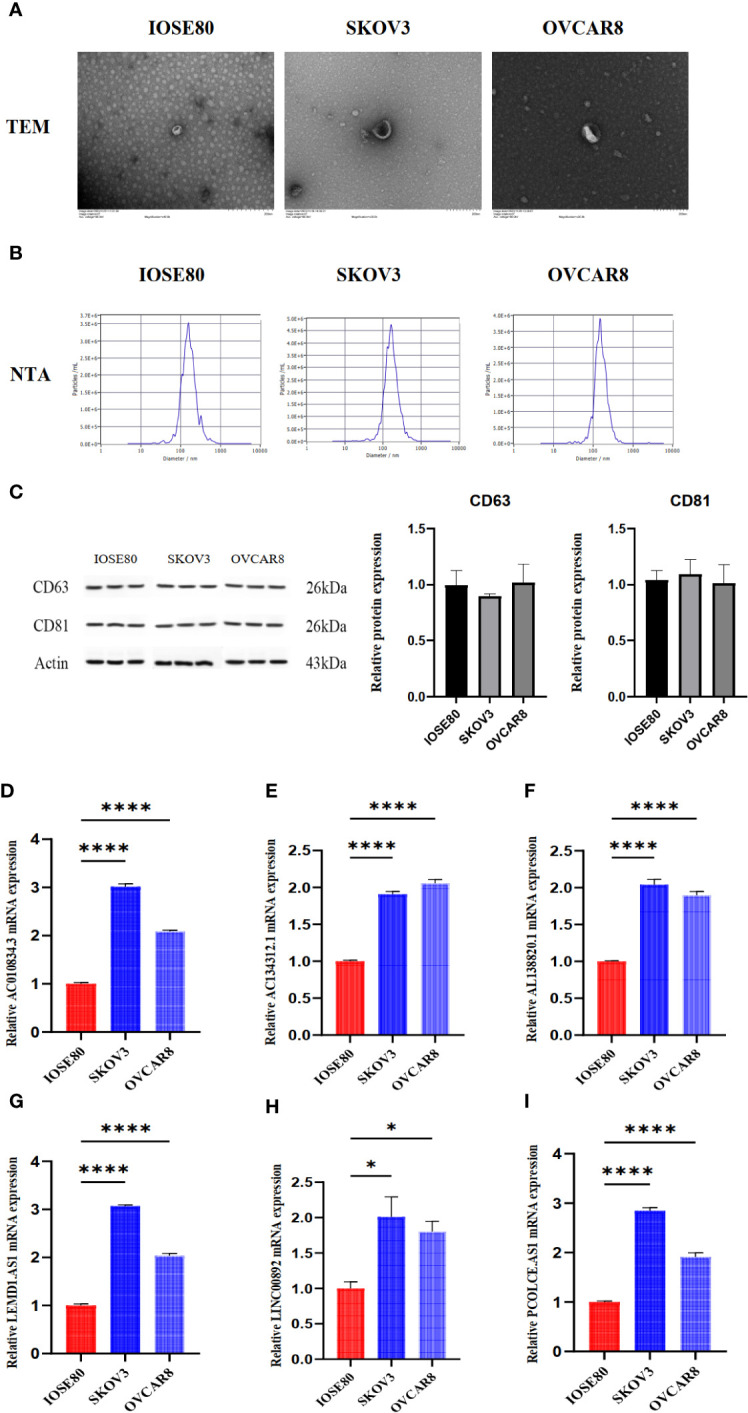
The detection of exosomes characteristics and lncRNA expression in exosomes from IOSE80 cells, SKOV3 cells, and OVCAR8 cells. **(A)** The results of TEM for exosomes. **(B)** The results of NTA for exosomes. **(C)** The expression of CD63 and CD81 in exosomes was detected using WB. **(D–I)** The expression of lncRNA in exosomes was measured using RT-PCR. The RT-PCR and WB experiments were independently repeated three times, with three replicate wells for each independent repetition. *P < 0.05, ****P < 0.0001.

We used ultracentrifugation to purify exosomes from the supernatants of SKOV3 cells, IOSE80 cells, and OVCAR8 cells and identified the exosomes. TEM analysis revealed that the exosomes are microvesicles with a diameter range of 30 to 150 nm, which are globular and have a typical cup shape (**Figure 9A**). NTA showed that the diameter of exosomes concentrated at 100 nm (**Figure 9B**). The biomarkers of exosomes (CD81, CD63) were detected by Western blotting (**Figure 9C**). Real-time quantitative PCR results showed the different expression of lncRNA in exosomes from normal ovarian epithelial cells and OC cells (**Figures 9D–I**).

## Discussion

Exosomes, derived from malignant tumor cells, serve as communicators for intercellular communication (45). Identification of genetic material signatures in exosomes is expected to be a potential marker to improve the clinical prognosis of OC patients (46, 47). In our paper, we integrated exosome-related lncRNA to construct ERLS aiming to evaluate the prognosis and immunotherapy response of OC patients. The risk score for ERLS was calculated by multiplying the expression levels of 20 lncRNAs by the corresponding coefficients. Based on the ERLS risk score, OC patients were divided into high- and low-risk groups. Compared to the low-risk group, the high-risk group has a worse prognosis. Multivariate COX regression analysis showed that the ERLS was an independent risk factor for prognosis. With regards to predicting immunotherapy response, the ERLS was able to distinguish the benefit of anti-PD1 or anti-CTLA4 immunotherapy. The ERLS combined with the expression of PDL1 or CTLA4 can more accurately predict the prognostic risk of OC patients. Patients with low expression of PDL1 or high expression of CTLA4 and low ERLS risk score had the best prognosis, while those with low PDL1 or CTLA4 expression and high ERLS risk score had the worst prognosis.

Exosome-related lncRNA and prognostic lncRNA were used to identify candidate lncRNAs. Subsequently, a total of 36 significant lncRNAs were screened using a combination of the stepCox(forward) and Ridge arithmetic. A multivariate Cox regression model was employed to construct the ERLS model, which includes 20 lncRNAs. Research indicates that these lncRNAs have also been utilized in prognostic models for ovarian cancer (48–52). Compared to other models (53, 54), we used more machine learning algorithms to make our model more robust. Survival analysis showed a worse prognosis in the high ERLS group. Furthermore, multivariate COX regression analysis showed that the ERLS was an independent prognostic factor for patients with OC. The ROC areas for 1-, 3-, and 5-years were 0.758, 0.816, and 0.827 in the entire TCGA datasets, respectively. The results of external validation showed that the AUC values of 1-, 3-, and 5-years were 0.536, 0.548, and 0.722, respectively. In particular, it has more advantages in long-term survival prediction. In addition, compared to other models in the entire TCGA database, the ERLS is competitive. These results suggest that the ERLS can identify prognostic risk in OC patients, indicating that the ERLS has great potential for clinical application.

Exosome-related lncRNA are key messenger molecules that regulate immune responses in the tumor microenvironment (55). In the tumor microenvironment, information continues to flow between immune cells and cancer cells through these RNAs, and inhibition of immune cell function induces the formation of an immunosuppressive tumor microenvironment, which affects the response to immunotherapy (56). Therefore, we investigated immune cell infiltration in high- and low-risk groups based on the xCell and ssGSEA packages.

The results showed that higher levels of DC, M1 macrophages, CD8 T cells, CD4 memory T cells, and Th2 cells were in the low-risk group, and this result was further verified in the ssGSEA package. The analysis of immune function differences between the high-risk group and the low-risk group showed that different levels of immune cell infiltration promoted the activation of the cytolytic activity, inflammation-promoting, T cell co-stimulation pathway, indicating that the low-risk group had a higher level of anti-inflammatory tumor activity. We performed GO and KEGG analysis on the genes that were different between the two groups. The results of the KEGG analysis showed that the differentially expressed genes were mainly involved in the MAPK signaling pathway and the PI3K-Akt signaling pathway. As reported in the literature, the abnormality of the MAPK signaling pathway or the PI3K-Akt signaling pathway can cause cancer, which in turn affects the function of immune cells (57).

The ratio of infiltration of immune cells in the tumor microenvironment will limit the effectiveness of immunotherapy. PD1, PDL1, and CTLA4 are commonly used immune checkpoints, but the overall response rate to immune checkpoint inhibitors is not high (58). Therefore, we assessed the potential of the ERLS to predict response to immunotherapy. The TIDE tool was used to assess the potential for tumor immune escape and predict the immunotherapy response in OC patients. Our results showed that there were no differences in the TIDE scores between the two groups, but the TIDE tended to be higher in the high-risk group, suggesting that the high-risk group may have less benefit from immunotherapy. Furthermore, we verified the ability of the ERLS model to predict the response to immunotherapy through the expression of IPS in the TICA database, TMB, and immune checkpoints. The results showed that the low-risk group may benefit more from immunotherapy, suggesting that the ERLS model has the potential to predict response to immunotherapy.

Subsequently, we found that the expression of the PDL1 or CTLA4 genes could not effectively assess the prognostic risk in the entire TCGA cohort which had been divided into high- and low-risk groups based on the median expression of PDL1 or CTLA4 in the training set. This is not consistent with other studies (59–62) and may be attributed to variations in the thresholds set for PDL1 or CTLA4 expression. It should be noted that the use of the median division threshold in this study was necessary to maintain consistency with the ERLS threshold division method. Nonetheless, in cases where there is a similar expression of PDL1 or CTLA4, the prognostic risk of OC patients cannot be well differentiated. Therefore, we implemented a combination of PDL1 or CTLA4 expression and the ERLS score to evaluate the prognosis. It was found that the ERLS model had a good ability to discriminate a prognosis in the case of similar expression of PDL1 or CTLA4. Notably, patients with low expression of PDL1 or CTLA4 and high ERLS had the worst survival. Patients with low expression of PDL1 or high expression of CTLA4 and low ERLS have the best prognosis. This suggests that the combination of PDL1 or CTLA4 and ERLS differentiates prognosis and optimizes clinical management of OC.

In addition, we detected the expression of some lncRNAs in exosomes derived from IOSE80, SKOV3, and OVCAR8 cells. The results showed that compared to IOSE80 cells, exosomes from SKOV3 and OVCAR8 cells had higher expression of AC134312.1, AC010834.3, LEMD1.AS1, PCOLCE.AS1, LINC00892, and AL138820.1. Research has shown that high expression of lncRNA is associated with the proliferation of ovarian cancer cells, activation of the PI3K-AKT pathway, T cell activation, and immune infiltration in the tumor microenvironment (63–66). However, this trend was not observed in OC tissues from the TCGA datasets except LINC00892. We speculate that this trend may be due to more lncRNAs being encapsulated in exosomes and secreted into the extracellular environment, leading to lower expression in OC tissues. Unfortunately, research on this phenomenon has not yet been explained. Additionally, these lncRNAs were employed to structure OV prognostic models. AC134312.1 is related to the Wnt signaling pathway and T cell receptor pathway (49). LINC00892, as one of the immune-related lncRNAs, has been used in OV prognostic models (51). PCOLCE.AS1 has been confirmed to be related to prognosis in breast cancer (67), but its role in ovarian cancer has not yet been determined. AC010834.3 and AL138820.1 lncRNAs have not yet been studied in the context of OV. Our initial findings of these lncRNAs being highly expressed in ovarian cancer-derived exosomes provide direction for future research.

In a word, considering the importance of exosome-related lncRNAs in the progression of OC, we integrated bioinformatics and machine learning algorithms to identify exosome-related lncRNA signatures (ERLS) to assess the prognosis, immune cell infiltration, and response to immunotherapy. The ERLS model is a promising tool to optimize decision-making and monitoring regimens in individual OC patients. However, our research still has certain deficiencies. This article only constructs the ERLS model from the perspective of genetic data to evaluate the prognosis, immune microenvironment, and immunotherapy response of OC patients and has not been validated using cell lines and patient samples.

## Data availability statement

The original contributions presented in the study are included in the article/**Supplementary Materials**, further inquiries can be directed to the corresponding author/s.

## Author contributions

YC wrote the main manuscript text and performed the bioinformatics analysis. WL guided and modified the manuscript. YF and YC wrote the corresponding R language code. WZ and YZ retrieved and downloaded the data. XW, DZ, and ZZ prepared the Figures. DZ, WL, and ZZ revised the manuscript. YC and WZ completed all experiments. All authors contributed to the article and approved the submitted version.
