# Supplementary figures and images for: An exosome-derived lncRNA signature identified by machine learning associated with prognosis and biomarkers for immunotherapy in ovarian cancer
Source: Front Immunol. 2024 Feb 9;15:1228235. doi: 10.3389/fimmu.2024.1228235 (PMC10884316; doi:10.3389/fimmu.2024.1228235)

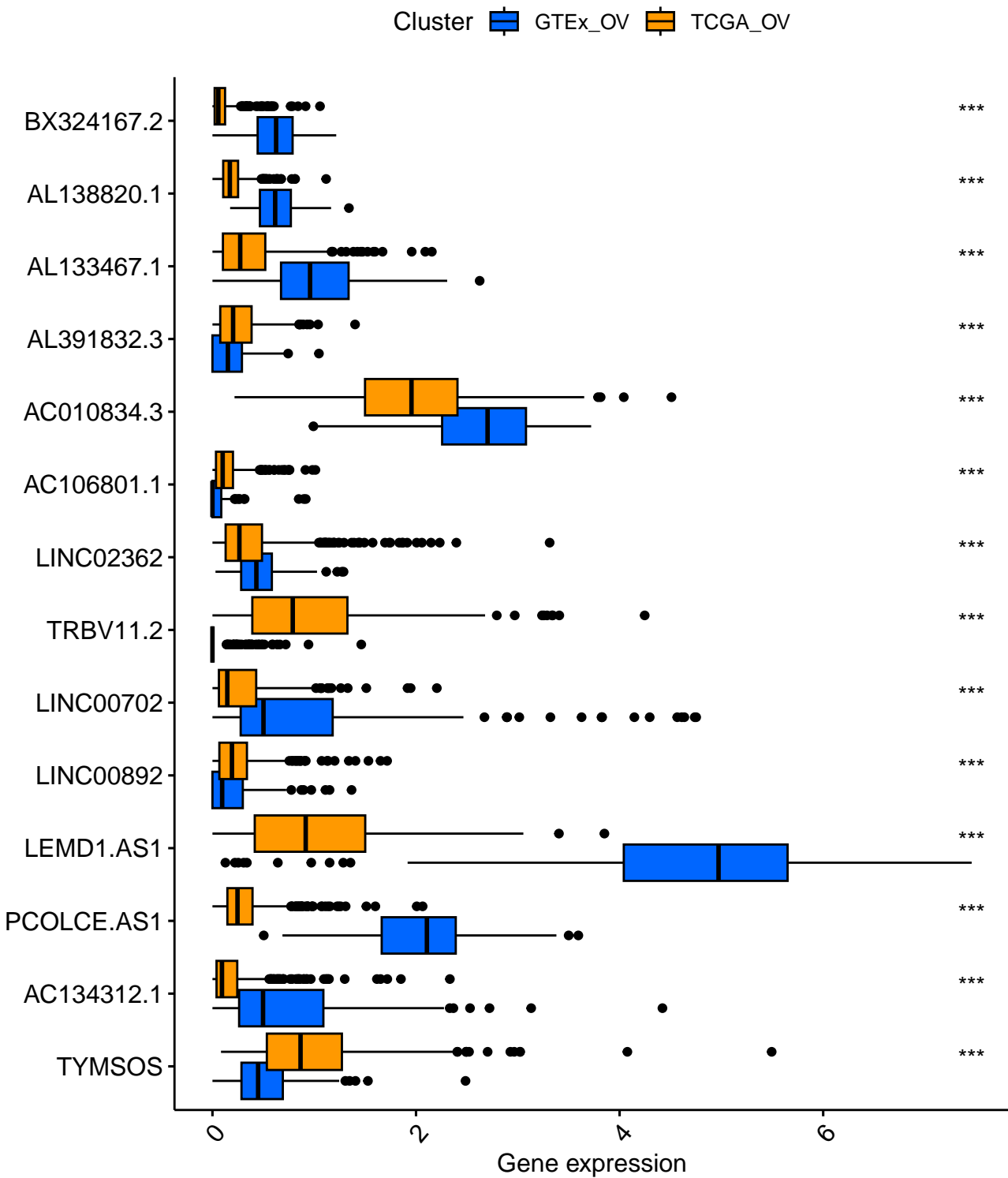

Supplement: Supplementary file 1 [file DataSheet_1.pdf]
