# Supplementary material for: An exosome-derived lncRNA signature identified by machine learning associated with prognosis and biomarkers for immunotherapy in ovarian cancer
Source: Front Immunol. 2024 Feb 9;15:1228235. doi: 10.3389/fimmu.2024.1228235 (PMC10884316; doi:10.3389/fimmu.2024.1228235)
Supplement: Supplementary file 3 [file DataSheet_3.docx]

To identify potential biomarkers for OC, candidate lncRNAs were further screened using 10 machine learning algorithms and 117 algorithm combinations. In the training dataset, 10 machine learning algorithms and 117 algorithm combinations were employed to identify the optimal algorithm combinations based on 10-fold cross-validation, which was verified in the verification data set. The selection of the best algorithm combinations was based on Harrell's consistency index (C index) in the validation dataset. 10 machine learning algorithms include Random Survival Forest (RSF), Lasso, Elastic Net (Enet), Ridge, Generalized Boosted Regression (GBM), Stepwise Cox, CoxBoost, Cox Partial Least Squares Regression (plsRcox), Supervised Principal Components (SuperPC), and survival support vector machine (survival-SVM). The RSF model was implemented via the randomForestSRC package. RSF had two parameters ntree and mtry, where ntree represented the number of trees in the forest and mtry was the number of randomly selected variables for splitting at each node. We set ntree to 1000 and mtry to its default value. All the pairs of (ntree, mtry) were formed and the one with the best C-index value was identified as the optimized parameters. The Enet, Lasso, and Ridge were implemented via the glmnet package. The regularization parameter, lambda, was determined by 10-fold cross-validation, whereas the L1-L2 trade-off parameter, α, was set to 0-1 (interval =0.1). The stepwise Cox model was implemented via survival package. A stepwise algorithm using the AIC (Akaike information criterion) was applied, and the direction mode of stepwise search was set to "both", "backward", and "forward", respectively. The CoxBoost model was implemented via CoxBoost package, which is used to fit a Cox proportional hazards model by componentwise likelihood-based boosting. For the CoxBoost model, we used 10-fold cross-validation routine optimCoxBoostPenalty function to first determine the optimal penalty (amount of shrinkage). Once this parameter was determined, the other tuning parameter of the algorithm, namely, the number of boosting steps to perform, was selected via the function cv.CoxBoost. The dimension of the selected multivariate Cox model was finally set by the principal routine CoxBoost. The plsRcox model was implemented via plsRcox package. The cv.plsRcox function was used to determine the number of components requested, and the plsRcox function was applied to fit a partial least squares regression generalized linear model. The SuperPC model was implemented via superpc package, is a generalization of principal component analysis, which generates a linear combination of the features or variables of interest that capture the directions of largest variation in a dataset. The superpc.cv function used the10-fold cross validation to estimate the optimal feature threshold in supervised principal components. To avoid problems with fitting Cox models to small validation datasets, it uses the "pre-validation" approach. The GBM model was implemented via the gbm package. Using the 10-fold cross validation, the cv.gbm function selected index for number trees with minimum cross-validation error. The gbm function was used to fit the generalized boosted regression model. The survival-SVM model was implemented via survivalsvm package. Subsequently, the selection of important variables was achieved using the stepAIC function in the MASS package. The Akaike Information Criterion (AIC) is used to compare models, which takes into account the statistical fit of the models and the number of variables used for the fit. The regression model with a small AIC value should be selected first, which shows that the model has obtained a sufficient fitting degree with few parameters. Finally, we constructed the ERLS model using a multivariate COX regression, and the risk score was constructed with the following formula：Risk score= $Risk score=\sum_{i=1}^{n} \left( coefi*Expi \right)$, Expi indicated the expression level for each exosome-related lncRNA, and Coei indicated the corresponding Cox regression coefficient.
